# Abnormal laughter-like vocalisations replacing speech in primary progressive aphasia

**DOI:** 10.1016/j.jns.2009.04.021

**Published:** 2009-09-15

**Authors:** Jonathan D. Rohrer, Jason D. Warren, Martin N. Rossor

**Affiliations:** Dementia Research Centre, Department of Neurodegenerative Disease, Institute of Neurology, University College London, Queen Square, London, WC1N 3BG, UK

**Keywords:** Primary progressive aphasia, Frontotemporal lobar degeneration, Laughter

## Abstract

We describe ten patients with a clinical diagnosis of primary progressive aphasia (PPA) (pathologically confirmed in three cases) who developed abnormal laughter-like vocalisations in the context of progressive speech output impairment leading to mutism. Failure of speech output was accompanied by increasing frequency of the abnormal vocalisations until ultimately they constituted the patient's only extended utterance. The laughter-like vocalisations did not show contextual sensitivity but occurred as an automatic vocal output that replaced speech. Acoustic analysis of the vocalisations in two patients revealed abnormal motor features including variable note duration and inter-note interval, loss of temporal symmetry of laugh notes and loss of the normal decrescendo. Abnormal laughter-like vocalisations may be a hallmark of a subgroup in the PPA spectrum with impaired control and production of nonverbal vocal behaviour due to disruption of fronto-temporal networks mediating vocalisation.

## 1 Introduction

Patients with dementia commonly exhibit a range of abnormal nonverbal vocalisations in the later stages of the illness: examples include screaming, singing, chanting, humming and grunting [1]. Many such patients have evidence of severe, widespread cognitive deficits. Abnormal vocalisations in dementia tend to be simple and stereotyped and may indicate the release of ‘automatic’ vocal behaviours that require less neural organisation than speech. However, the relationship between abnormal vocalisations and impairments of speech output has not been defined, nor has it been established whether different kinds of vocalisations are associated with specific diagnoses.

Relatively early and selective breakdown of speech output is a feature of the primary progressive aphasias (PPA), a clinically, radiologically and pathologically diverse group of diseases. Three canonical clinical syndromes have been described: progressive non-fluent aphasia (PNFA) where there is impairment of articulation (commonly an apraxia of speech) and agrammatism; semantic dementia (SD) where there is progressive breakdown of conceptual knowledge; and the logopenic or phonological variant of PPA (LPA) where there is a decreased speech rate with word-finding pauses and anomia [2–4]. While reduction in spontaneous speech leading to mutism is integral to PNFA, all three syndromes may lead eventually to mutism via different mechanisms [5]. In SD, degradation of language content is associated with increasingly empty speech until the patient is reduced to using a few stock phrases or single words, and in LPA there is increasing anomia and decreased speech rate. PPA is generally considered part of the frontotemporal lobar degenerations (FTLD) and there is overlap with behavioural variant frontotemporal dementia (bvFTD). Some patients in this overlap group develop impaired generation of verbal thought or “dynamic aphasia” [6].

Here we describe a group of patients with progressive aphasia who exhibited abnormal laughter-like vocalisation (LLV) that increasingly replaced speech as the disease evolved, until ultimately LLV was the only extended utterance produced by these patients.

## 2 Patient characteristics

In the period between 1996 and 2007, ten patients were ascertained via the Specialist Cognitive Disorders Clinic of the National Hospital for Neurology and Neurosurgery with a clinical syndrome of reduced speech output accompanied by excessive LLV. All patients fulfilled consensus criteria for PPA or FTLD at presentation [2,7,8]. The clinical, neuropsychological, radiological and pathological features of the cases in this series are summarised in Table 1. The most common PPA phenotype in this series was PNFA. All patients ultimately became mute with LLV as their only vocal output. Several patients were echolalic before complete mutism supervened. None of the patients had signs of corticobulbar dysfunction or other clinical evidence of motor neuron disease. The characteristics of the LLV were similar in all cases. As patients became mute they were noted to develop increasingly prominent LLV, often continuing intermittently for a number of minutes. LLV was intrusive and often inappropriate to emotional context (for example, one patient laughed after his wife told him their dog had died). When assessed in clinic, patients would commonly laugh in response to any question or other attempt to engage them in conversation. This laughter was abnormal in quality, often shrill and uncoordinated, and did not have the ‘infectious’ property of normal laughter. Behavioural changes were present in 7/10 cases (Table 1). Asymmetric (predominantly left-sided) cerebral atrophy was present in 7/9 patients who underwent brain imaging: atrophy was most severe in the frontal and anterior temporal lobes, and extended posteriorly to involve the parietal lobe in 4/9 cases. Post mortem examination in three cases revealed tau positive neuronal inclusions in one case and tau-negative, TDP-43 positive inclusions in the other cases [9].

During the period these cases were ascertained, a total of 210 patients with a diagnosis of PPA presented to the clinic. Based on this series, the estimated prevalence of LLV in PPA is therefore of the order of 10/210 or 5%. This may be an underestimate as many patients were not followed as their disease progressed. Over the same time period, no patients attending the clinic with an alternative neurodegenerative diagnosis (including approximately 650 patients with a clinical diagnosis of Alzheimer's disease and 170 patients with a clinical diagnosis of Dementia with Lewy Bodies) exhibited LLV, suggesting that the prevalence of the syndrome in other degenerative diseases (though undefined) is very low.

## 3 Analysis of laughter-like vocalisations

Normal laughter has a characteristic acoustic signature and quantitative data are available [10]. A laugh is a series of short vowel-like “laugh notes” (most commonly ‘hah’ or ‘hoh’) that occur at regular intervals (i.e. with a relatively fixed inter-note interval) and with relatively constant duration. Inter-note interval and note duration are positively correlated, consistent with a common production mechanism. The individual notes in a laugh sequence have a spectrogram that is similar whether the note is played forward or backward (temporal symmetry), whereas the laugh sequence as a whole is temporally asymmetric due to a characteristic decrescendo of note amplitude as the laugh progresses. In addition to these acoustic features, normal laughter is context-sensitive, typically punctuating conversational pauses and modulated by social cues (e.g., it is more likely to occur in response to the laughter of others).

**Fig. 1:**
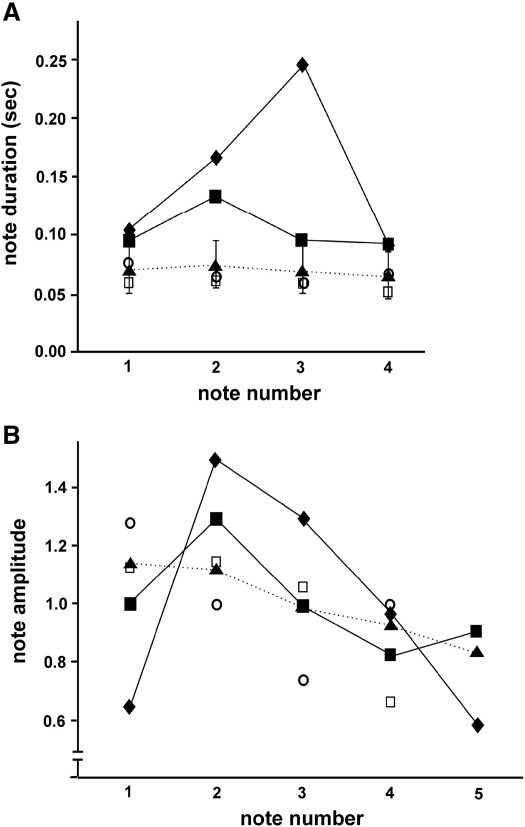
A) Mean laugh note duration (s) for the first four notes of pooled laughter samples from Case 2 (diamonds), Case 5 (squares), disease control cases without abnormal laughter-like vocalisations (patient with familial Alzheimer's disease, open circles; patient with behavioural variant FTLD, open squares), and a healthy control sample (triangles and dotted line, with error bars representing mean standard deviation) of 28 healthy female subjects (data from Provine and Yong [10]). B) Trends in mean laugh note amplitude for pooled laughter-like vocalisation samples from Case 2, Case 5, disease control cases without the syndrome and a control sample of 51 healthy subjects (data from Provine and Yong [10]). Symbols as in (A). Amplitude units are arbitrary; samples have been normalised to a mean amplitude of 1.

We analysed recorded samples of LLV from two patients in this series (Cases 2 and 5) using an acoustic software package (PRAAT, [11]). Both patients were mute at the time of recording. Data are summarised in Fig. 1 and in Appendix A and acoustic samples of patients' vocalisations are available as supplementary material online in Appendix A). Qualitatively, LLV in both these and the other cases was dysrhythmic and fragmentary, and tended to occur spontaneously in frequent short bursts lasting only a few notes and not influenced by conversational or other social cues. Quantitatively, in comparison to normative female data [10], the LLV of both Cases 2 and 5 had abnormally prolonged and variable laugh note durations (Fig. 1A) and inter-note intervals. Neither case exhibited the normal decrescendo of laugh note amplitude across the laugh sequence (Fig. 1B). In addition, the individual laugh notes in Case 2 had asymmetric (non-reversible) temporal envelopes. Together these acoustic characteristics indicate that LLV in these cases lacked the highly stereotyped and rhythmic structure of normal laughter.

To further assess the specificity of the syndrome, we analysed random samples of laughter from two female patients with a diagnosis of a neurodegenerative dementia (one aged 40 with familial Alzheimer's disease on the basis of a presenilin 1 mutation; the other aged 45 with a clinical diagnosis of bvFTD) who did not have the clinical syndrome of abnormal laughter. Qualitatively, laughter in these patients tended to occur in conversational pauses and was broadly appropriate to social or emotional context (though the patient with bvFTD laughed in the context of fatuous humour). Quantitative analysis (Fig. 1 and Appendix A) revealed that laughter in the disease control patients shared many features with the laughter of healthy control subjects and did not resemble the laughter of patients with the abnormal laughter syndrome.

## 4 Discussion

Here we have characterised a syndrome of abnormal laughter-like vocal output accompanying mutism in a series of patients with PPA. While LLV frequently occurred in a conversational setting, it was not modulated by conversational pauses or other cues and there was no evidence the patients recognised their own laughter as abnormal. LLV was not associated with features of corticobulbar dysfunction or with abnormal crying or other overt emotional displays, suggesting the LLV syndrome is pathophysiologically distinct from the pathological laughter that frequently accompanies diseases with involvement of the corticobulbar tracts. While other behavioural changes did develop in a majority of cases, this was not inevitable (Table 1). Taken together, this evidence suggests that the patients in this series had a specific breakdown of the modulatory controls that govern laughter onset and offset, rather than simply a compensatory response or an effect of general fatuity or emotionalism. The LLV comprised short bursts (typically only a few notes) that became increasingly frequent as the disease evolved and spontaneous speech diminished. In addition, echolalia developed in four of the cases in this series. This is consistent with increasing automaticity of vocal motor programmes associated with degradation of propositional speech. Although LLV in our patients ‘replaced’ propositional speech, due to their highly disorganised nature the vocalisations did not serve any useful communicative function, and lacked any propensity to ‘infect’ the listener. The acoustic signature of LLV in these cases was dysrhythmic and lacked the hallmark decrescendo of normal laughter. Disorganisation of phoneme formation with substantial variability characterises apraxia of speech in progressive aphasia [12], the hypothesis being that this is due to a deficit at the level of planning of articulation [13]. Analogously, the abnormal structure of laughter in our cases may result from an ‘apraxia’ of nonverbal vocal output, in a similar sense of a deficit at the stage of planning. The case numbers here were small and corroboration of these findings with quantitative laughter analysis in larger prospective studies is required. However, we propose that LLV in these PPA cases has the core features of contextual insensitivity, automaticity, and motor disorganisation. Although these quasi-periodic, brief vocalisations bear some superficial resemblance to normal laughter, it is clear that both acoustically and contextually they constitute an abnormal form of vocal output that is far removed from the highly organised social vocal behaviour that we generally recognise as laughter.

The abnormal LLV syndrome we have characterised is also unlike other forms of pathological laughter in neurological disease. Pathological laughter can be defined as involuntary laughter in the absence of a congruent emotion, and has a variety of disease associations [14,15], including pseudobulbar palsy in motor neuron disease and multiple sclerosis [16,17], epilepsy (‘gelastic’ seizures arising from various locations: [18,19], ‘fou rire prodromique’ in which uncontrollable laughter heralds an ischaemic event [20,21], and other focal lesions of the brainstem [22,23], frontal [24–26] and temporal [27] lobes. In the context of corticobulbar dysfunction, pathological laughter and crying are often associated and both may occur spontaneously or in response to minor or apparently irrelevant stimuli, consistent with loss of descending inhibitory controls over brainstem motor pattern generators. While abnormal vocalisations of various kinds are well recognised in dementia [1], these vocalisations have not been fully characterised. In a series of 12 patients with presumed Alzheimer's disease or vascular dementia, abnormalities of ‘noise-making’ in dementia were classified as (a) persistent screaming, (b) perseverative vocalisation, (c) continuous chattering, muttering, singing or humming, and/or (d) swearing, grunting and ‘bizarre’ [1]. Of those twelve patients, five patients had little or no speech, however none was described as having inappropriate laughter, whereas a study looking specifically at patients with Alzheimer's disease found that 14% of patients had either laughing or mixed laughing and crying episodes [28]. A number of previously described cases of abnormal laughter in the context of degenerative disease have had mutism associated with damage involving frontotemporal or fronto-subcortical circuits. One patient had features of both PNFA and a corticobasal syndrome [29] while others had CJD [30,31]. Another patient developed excessive laughter in the context of FTLD [32]: in that case, laughter was not appropriate to context and was not associated with any affective change, and brain imaging revealed bilateral but asymmetrical (predominantly left-sided) fronto-temporo-parietal atrophy. The pervasive occurrence of laughter effectively replacing speech has not been described with other processes causing abnormal laughter, suggesting that the pathophysiological basis of LLV in patients with progressive speech output breakdown and mutism may be distinct from abnormal laughter in other diseases. This is supported by the analysis of laughter in other disease states presented here (Fig. 1).

Although laughter has attracted the attention of clinicians and scientists for many years [20,33–35], detailed studies of the brain basis of laughter in health or disease are comparatively few. Human laughter is a complex nonverbal vocal and social behaviour with a number of functionally separable motor, affective and cognitive subprocesses [10,35]. With the advent of functional brain imaging techniques, the functional neuroanatomy of laughter has been studied in cognitively-normal individuals. These studies have delineated a widely-distributed cerebral ‘laughter network’ comprising a number of cortical and sub-cortical areas (e.g. [36–40]; reviewed in [41]). The perception of laughter involves areas in both cerebral hemispheres including the amygdalae, insulae and anterior and posterior non-primary auditory areas, and primary somatosensory and pre-motor regions, with evidence for rightward functional asymmetry [41]. Partly on technical grounds, the production of laughter has been less well-studied than its perceptual mechanisms, however the expression of affective states is mediated by an overlapping cerebral network including bilateral pre-motor areas [36], bilateral basal temporal areas (temporal poles, parahippocampal gyri, hippocampi and amygdalae) [40] and bilateral insulae and somatosensory regions [37]. The brain mechanisms that govern the perception and production of positive vocal emotions (including laughter) may be functionally linked via mirror neurons linked to the human dorsal cortical auditory pathway [38,42]. This pathway is likely to have a generic role in the preparation of responses to speech, nonverbal vocalisations and other complex sounds: while there is differential hemispheric selectivity for verbal and nonverbal sounds, functional imaging evidence in human subjects supports bi-hemispheric activation of the pathway with emotional vocalisations [42]. The cortical networks for laughter perception and production encompass a number of key brain regions implicated in the control of human social behaviour more generally [43], and commonly damaged in FTLD [12]. Regions of maximal atrophy in the present cases overlap frontal, temporal and parietal regions previously implicated in laughter production (Table 1). However, the present series does not allow determination of a specific structural correlate of LLV: similar patterns of atrophy are also observed in patients with PPA who do not develop the LLV syndrome. Similarly, the syndrome is unlikely to have a specific molecular substrate based on limited pathological evidence (Table 1).

Based on the clinical and acoustic and evidence presented here, we propose that some patients with PPA develop abnormal LLV as a specific functional derangement of fronto-temporal networks for the regulation and production of nonverbal vocalisations. Highly organised behaviours such as laughter are mediated by distributed cerebral networks [10,35–41], and it is therefore plausible that such behaviours are vulnerable to diffuse, network level disease processes (such as PPA). The distributed nature of the disease process might affect cerebral mechanisms that control the expression of behaviour (leading to abnormal regulation or ‘liberation’ of behaviour that is contextually inappropriate), as well as cerebral mechanisms that programme motor commands (leading to abnormally executed or ‘dyspraxic’ behaviour). These regulatory and executive mechanisms are likely to require the interaction of frontal and temporal lobe circuits: the development of abnormal nonverbal vocalisations in FTLD may be pathophysiologically analogous to the expression of abnormal non-vocal stereotypical behaviours [44], which are likely to result from damage to fronto-temporal networks that govern other kinds of complex voluntary actions. The LLV syndrome is likely to be more frequent as a feature of disease evolution in a subgroup of patients with PPA and predominant involvement of vocal output pathways. It remains to be established whether the pattern of damage is stochastic, or whether the laughter syndrome emerges from the correlated involvement of functionally linked cortical areas. This ‘gelastic dementia’ syndrome might be considered a prototypical disorder of non-verbal vocal and social behaviour in neurodegenerative disease.

## Figures and Tables

**Table 1:** Clinical, neuropsychological and brain imaging features of cases with abnormal laughter-like vocalisations.

Case no.	Sex	Age at onset	Diagnosis	Anomia	Apraxia of speech	Single word comprehension impairment	Echolalia	Other behavioural symptoms	Brain imaging⁎				Pathology
									Asymm	Frontal lobe	Temporal lobe	Parietal lobe	
1	M	52	PNFA	+	+	−	−	Social disinhibition, loss of empathy	L=R	++	++	−	FTLD-TDP
2	F	54	PNFA	+	+	−	+	Apathy, sweet tooth	L>R	++	++	+	n/a
3	M	56	Atypical SD	+	−	+	+	Aggression, disinhibition, obsessionality, hyperphagia	L>R	++	++	+	FTLD-tau (Pick's disease)
4	F	62	Dynamic aphasia (bvFTD)	+	−	−	−	Apathy, sweet tooth, rituals	L>R	++	++	+	FTLD-TDP
5	F	60	PNFA	+	+	−	−	None	L>R	++	++	−	n/a
6	M	45	PNFA	+	+	−	−	Loss of empathy, sweet tooth	L>R	++	++	+	n/a
7	F	46	PNFA	+	+	−	−	None	L>R	++	++	−	n/a
8	F	52	PNFA	+	+	−	−	None	L=R	++	++	−	n/a
9	F	56	Dynamic aphasia (bvFTD)	+	−	−	+	Social withdrawal, fixed routines and rituals, utilisation behaviour	L>R	++	++	−	n/a
10	M	58	Dynamic aphasia (bvFTD)	+	−	−	+	Personality change, anxiety, obsessionality	n/a	n/a	n/a	n/a	n/a

Key: ⁎MRI in all except Case 1 (CT) and Case 10; n/a = not available; for clinical and pathological diagnoses: bvFTD = behavioural variant frontotemporal dementia, FTLD = frontotemporal lobar degeneration, PNFA = progressive nonfluent aphasia, SD = semantic dementia, TDP = TAR DNA binding protein; f or speech and language features: + = present, − = absent; for imaging, Asymm = asymmetry, L = left, R = right, ++ = moderate atrophy, + = mild atrophy, − = no atrophy (areas within the left hemisphere).

## Appendix A

Supplementary table Acoustic characterisation of patients with PPA and abnormal laughter versus disease controls.
