# Supplementary material for: Abnormal laughter-like vocalisations replacing speech in primary progressive aphasia
Source: J Neurol Sci. 2009 Sep 15;284(1-2):120–3. doi: 10.1016/j.jns.2009.04.021 (PMC2729814; doi:10.1016/j.jns.2009.04.021)
Supplement: Supplementary file 1 — Supplementary table Acoustic characterisation of patients with PPA and abnormal laughter versus disease controls. [file mmc1.doc]

**Supplementary Table**

**Acoustic characterization of patients with PPA and abnormal laughter versus disease controls**

|  |  | **N1** | **N2** | **N3** | **N4** | **N5** | **N6** | **N7** | **N8** | **MEAN** | **SD** |
| --- | --- | --- | --- | --- | --- | --- | --- | --- | --- | --- | --- |
| **CASE 2** |  |  |  |  |  |  |  |  |  |  |  |
| **Sample 1** | **Amp** | 1.25 | 1.15 | 0.76 | 0.28 | 0.73 | 1.84 |  |  | **1.00** | **0.44** |
|  | **ND** | 0.072 | 0.115 | 0.16 | 0.053 | 0.109 | 0.409 |  |  | **0.153** | **0.131** |
|  | **INI** | 0.153 | 0.154 | 0.403 | 0.085 | 0.462 |  |  |  | **0.251** | **0.169** |
| **Sample 2** | **Amp** | 0.64 | 1.27 | 1.42 | 1.14 | 0.53 |  |  |  | **1.00** | **0.39** |
|  | **ND** | 0.049 | 0.09 | 0.115 | 0.106 | 0.115 |  |  |  | **0.095** | **0.028** |
|  | **INI** | 0.118 | 0.117 | 0.131 | 0.267 |  |  |  |  | **0.158** | **0.073** |
| **Sample 3** | **Amp** | 0.21 | 1.63 | 1.20 | 0.96 |  |  |  |  | **1.00** | **0.60** |
|  | **ND** | 0.184 | 0.292 | 0.47 | 0.107 |  |  |  |  | **0.263** | **0.157** |
|  | **INI** | 0.197 | 0.588 | 0.47 |  |  |  |  |  | **0.418** | **0.201** |
| **CASE 5** |  |  |  |  |  |  |  |  |  |  |  |
| **Sample 1** | **Amp** | 0.96 | 1.26 | 0.83 | 1.040 | 1.00 | 0.78 | 1.22 | 0.91 | **1.00** | **0.17** |
|  | **ND** | 0.110 | 0.139 | 0.093 | 0.091 | 0.114 | 0.101 | 0.116 | 0.111 | **0.109** | **0.015** |
|  | **INI** | 0.197 | 0.268 | 0.205 | 0.230 | 0.235 | 0.237 | 0.232 |  | **0.229** | **0.023** |
| **Sample 2** | **Amp** | 1.07 | 1.35 | 1.24 | 0.56 | 0.79 |  |  |  | **1.00** | **0.33** |
|  | **ND** | 0.075 | 0.125 | 0.095 | 0.089 | 0.076 |  |  |  | **0.092** | **0.020** |
|  | **INI** | 0.170 | 0.189 | 0.229 | 0.194 | 0.191 |  |  |  | **0.195** | **0.021** |
| **DISEASE**  **CONTROLS** |  |  |  |  |  |  |  |  |  |  |  |
| **bvFTD** |  |  |  |  |  |  |  |  |  |  |  |
|  | **Amp** | 1.12 | 1.15 | 1.07 | 0.66 |  |  |  |  | **1.00** | **0.23** |
|  | **ND** | 0.058 | 0.061 | 0.058 | 0.052 |  |  |  |  | **0.057** | **0.004** |
|  | **INI** | 0.168 | 0.177 | 0.179 |  |  |  |  |  | **0.175** | **0.006** |
| **AD** |  |  |  |  |  |  |  |  |  |  |  |
|  | **Amp** | 1.28 | 0.99 | 0.74 | 1.00 |  |  |  |  | **1.00** | **0.27** |
|  | **ND** | 0.076 | 0.063 | 0.059 | 0.071 |  |  |  |  | **0.067** | **0.008** |
|  | **INI** | 0.168 | 0.167 | 0.174 |  |  |  |  |  | **0.168** | **0.001** |

**Key:**

Amp = amplitude

N(x) = note (number)

ND = note duration

INI = internote interval

Values for ND and INI are in seconds; amplitude in arbitrary voltage units measured on digital wavefile and normalised to mean amplitude of 1 V peak-to-peak
